# Spinocerebellar ataxia in the Italian Spinone dog is associated with an intronic GAA repeat expansion in *ITPR1*

**DOI:** 10.1007/s00335-014-9547-6

**Published:** 2014-10-30

**Authors:** Oliver P. Forman, Luisa De Risio, Kaspar Matiasek, Simon Platt, Cathryn Mellersh

**Affiliations:** 1 Kennel Club Genetics Centre, Animal Health Trust, Kentford, Newmarket, Suffolk, CB8 7UU UK; 2 Neurology Department, Animal Health Trust, Kentford, Newmarket, Suffolk, CB8 7UU UK; 3 Section of Clinical & Comparative Neuropathology, Institute of Veterinary Pathology, Ludwig Maximilians University, Veterinarstr 13, 80539 Munich, Germany; 4 Department of Small Animal Medicine and Surgery, The University of Georgia College of Veterinary Medicine, 501 D.W. Brooks Drive, Athens, GA 30602 USA

## Abstract

Spinocerebellar ataxia in the Italian Spinone dog breed is characterised by a progressive gait abnormality that manifests from approximately 4 months of age. The disorder shows an autosomal recessive mode of inheritance, and affected individuals are usually euthanized by one year of age on welfare grounds due to an inability to ambulate. Using a homozygosity mapping technique with six cases and six controls, we mapped the disease locus to chromosome 20 of the canine genome. Linkage analysis across an extended pedigree confirmed the association, with microsatellite C20.374 achieving a maximal LOD score of 4.41. All five genes within the disease-associated interval were exon resequenced, although no exonic candidate mutations were identified. A targeted resequencing approach was therefore adopted to sequence the entire disease-associated interval. Analysis of the sequencing data revealed a GAA repeat expansion in intron 35 of *ITPR1*, which was homozygous in all cases and heterozygous in obligate carriers. Partial impairment of cerebellar *ITPR1* expression in affected dogs was demonstrated by immunohistochemistry. Given the association of *ITPR1* mutations with spinocerebellar ataxia (SCA) type 15 (also designated SCA16) in humans and that an intronic GAA repeat expansion has been shown to cause Friedreich ataxia, the repeat expansion is an excellent candidate for the cause of spinocerebellar ataxia in the Italian Spinone. This finding represents the first naturally occurring pathogenic intronic GAA repeat expansion in a non-human species and a novel mechanism for *ITPR1* associated spinocerebellar ataxia.

**Electronic supplementary material:**

The online version of this article (doi:10.1007/s00335-014-9547-6) contains supplementary material, which is available to authorized users.

## Introduction

Spinocerebellar ataxia in the Italian Spinone (SCAIS) is a progressive neurodegenerative disease characterised by hypermetria, particularly in the pelvic limbs, truncal ataxia and impaired balance. Clinical signs start to appear at four months of age and progress to a degree of dysfunction which leads to euthanasia of affected dogs at one year of age on average. The disease shows an inheritance consistent with an autosomal recessive mode. SCAIS was acknowledged in the veterinary literature as a brief clinical communication in 1996 (Wheeler and Rusbridge 1996), and has been reported anecdotally in a number of countries including Italy, UK, USA and Denmark. Cases are rare, with no new cases reported since the launch of a linkage based DNA test at the Animal Health Trust in 2008, enabling breeders to identify heterozygous carriers in their breeding lines.

In humans, clinically distinct types of spinocerebellar ataxias have been well characterised and numerically catalogued into autosomal recessive (SCAR1–12) and autosomal dominant (ADCA or SCA1–36) forms. For many of these forms disease-associated genes have been identified, allowing candidate gene studies to be undertaken in other species, an approach regularly adopted when studying canine disorders, and recently successfully used to identify the cause of a neonatal cerebellar ataxia in the Beagle dog (Forman et al. 2012). A range of mutation types have been associated with SCA in humans, including genomic deletions, duplications, point mutations and both intronic and exonic repeat expansions. Polyglutamine expansions are associated with several SCA types in humans including SCA1, 2, 3, 6, 7 and 17 (Higgins et al. 1996; Koide et al. 1999; Lindblad et al. 1996; Orr et al. 1993; Riess et al. 1997; Sanpei et al. 1996). Intronic repeat expansions account for SCA10 (Matsuura et al. 2000) and Friedreich ataxia (Campuzano et al. 1996), and 5′ and 3′ UTR repeat expansions are associated with SCA8 (Koob et al. 1999) and SCA12 (Holmes et al. 1999), respectively. The variety of mutation type is a key consideration when studying the molecular biology of spinocerebellar ataxia to ensure that causal mutations are not overlooked or misidentified.

In this investigation, we used a homozygosity mapping approach using six SCAIS cases and six controls to map the disease-associated locus. Results were followed up by exon resequencing of interval genes and subsequently targeted resequencing using a massively parallel sequencing technique in an attempt to elucidate the causal mutation. After identification of the candidate gene mutation, the expression of the gene product and its potential involvement with neurodegeneration was evaluated in serial sections of the cerebellum of three clinically and genetically confirmed SCA cases.

In this manuscript, we describe the identification of a novel intronic GAA triplet repeat expansion in the inositol 1,4,5-trisphosphate receptor, type 1 (*ITPR1*) gene. This represents the first naturally occurring pathogenic repeat expansion of its type in a non-human species and a novel mechanism for *ITPR1*-associated spinocerebellar ataxia.

## Materials and methods

### Sample collection

All DNA samples were collected from Italian Spinoni (IS) in the general pet dog population. We collected residual blood samples that were drawn as part of a veterinary procedure or buccal swab samples that were collected by owners or by veterinarians. DNA was collected from historic cases in the form of formalin fixed paraffin embedded (FFPE) cerebellum tissue. The clinical diagnosis of SCA was based on neurological examination by two board certified neurologists (LDR and SP) in four dogs and on revision of video footage and medical records in two other dogs.

### Homozygosity mapping and linkage analysis

Homozygosity mapping was performed using DNA from six SCAIS cases and six obligate carriers. A set of 325 genome-wide microsatellites was genotyped, of which 284 had a genotyping frequency of >90 %, and 260 were informative with a minor allele frequency >5 %. PCRs were performed in multiplex reactions of three to ten markers using fluorescently labelled forward primers for detection of products by capillary electrophoresis on ABI3100 genetic analysers. Genotypes were scored using Genemapper v4.0. Results were analysed initially by calculating the ratio of major allele homozygotes in the case versus control groups and also by visual inspection for a pattern suggestive of linkage. Positive results were followed up by performing linkage analysis. An extended IS pedigree was drawn using the Cyrillic software package and data exported for two-point linkage analysis using MLINK software.

### Sequencing

Exon resequencing and sequencing of an expanded GAA repeat allele were performed using a standard Sanger sequencing methodology, using Bigdye v3.1 chemistry. Sequencing products were separated on ABI3100 genetic analysers and data analysed using the Staden Gap4 software package.

Libraries for targeted resequencing of the disease-associated region were prepared using a custom SureSelect XT target enrichment kit (Agilent). Capture probes (baits) were design using the online tool e-array (https://earray.chem.agilent.com/earray/). Final libraries were quantified using the Kapa library quantification kit, and sequencing was performed at the Wellcome Trust Centre for Human Genetics, University of Oxford, on an Illumina HiSeq 2000. A 20 Gb dataset of 51 bp paired-end reads was produced. Reads were aligned to the CanFam2 genome sequence using BWA (Li and Durbin 2009), and SNP and INDEL calls made using GATK (McKenna et al. 2010). Read alignments were visualised using the Integrative Genome Viewer (IGV) (Thorvaldsdottir et al. 2013).

### Repeat expansion PCR

Long range PCR was performed to amplify GAA repeat alleles. Reactions consisted of 2.5 μl 10 × Qiagen LR reaction buffer, 1.25 μl dNTPs (10 mM each), 0.5 μM primers (forward: GGTGAGGAGCATGTTCTGGT; reverse: TGTCTCAGCGGTTGAATGTC), 2 μl Qiagen LongRange PCR Enzyme Mix and ultrapure water to a volume of 25 μl. Cycling parameters were 93 °C for 3 min, followed by 35 cycles of 93 °C for 15 s, 62 °C for 30 s and 68 °C for 3 min.

### Histology and immunohistochemistry

Donated IS brains were routinely fixed in 10 % neutral buffered formalin and trimmed in transverse and sagittal planes after longitudinal section in the midline. Amongst other levels, 0.3 mm serial sections of the cerebella and brainstem were embedded in paraffin after passing an ascending alcohol series and xylene immersion through an automatic tissue processor.

Our cerebellar staining panel included haematoxylin-eosin (HE), a modified Bielschowsky impregnation method (Erickson-Davis et al. 2010; Grimaldi and Manto 2012), and immunohistochemistry for synaptophysin (1:400, Syaptic Syystems GmbH, Goettingen, Germany), calbindin-D-28 K (1:1,000, Sigma, St. Louis, USA (Laure-Kamionowska and Maslinska 2009), microtubule associated protein-2 (MAP2; 1:1,000 (Resibois et al. 2007), inositol 1,4,5 tri-phosphate receptor-1 (ITPR1; 1:200; Linaris, Dossenheim, Germany (Miyata et al. 1999) and glial fibrillary acidic protein (GFAP; 1:500 Nobrega et al. 2013). Immunolabelling of Calbindin, MAP2 and ITPR1 employed microwave pretreatment in pH 6.0 citrate buffer. Primary antibody binding was visualised using Impress^®^ polymer technology (Linaris, Dossenheim, Germany) and a diaminobenzidine-based detection kit.

Assessment of the cerebellar morphology included lobulation and folia configuration, the subarachnoid space volume, cortical lamination pattern and cell density, as well as the morphology of foliary white matter, cerebellar roof nuclei, cerebellar peduncles, spinocerebellar tracts and the precerebellar pontine and olivary nuclei.

Purkinje cell features included their position and spatial distribution, the expression and subcellular distribution of differentiation markers, the dendritic tree configuration, the morphology of basket cell plexuses (Erickson-Davis et al. 2010) and the presence/absence of halo-like structures surrounding the PC (Yoshida et al. 2014).

## Results

### Clinical investigations

Neurological characteristics of SCAIS include a wide-based stance, spinocerebellar ataxia characterised by thoracic limb hypermetria (hyperextension), pelvic limb hyperflexion, truncal swaying, impaired balance, pendular nystagmus and absent menace response bilaterally. The remainder of the neurological examination was within normal limits. As the disease progressed intentional head tremor was observed and balance impairment deteriorated to the point that the dogs were unable to stand up and ambulate at approximately 1 year of age. Haematology, serum biochemistry and urinalysis were unremarkable in all affected dogs. MRI of the brain of two affected 8-month-old littermates (one male and one female) showed a subjective mild increase in sulcal widths and exaggerated definition of the folia in the rostral lobe of the cerebellum, suggesting grey matter loss. In addition, a cerebellar vermian defect was noted in the ventral portion of the cerebellum. However, these MRI changes were observed also on MRI of a clinically normal female littermate unaffected by the *ITPR1* mutation, and therefore their clinical significance remains dubious. MRI was repeated in the affected female dog 5.5 weeks after the first one; however, no changes were observed despite significant clinical deterioration. MRI of the cervical spine revealed no abnormalities in the two IS undergoing cervical MRI. The clinically normal individual subjected to MRI was followed up for several years by a veterinary neurologist and did not develop any clinical signs of spinocerebellar ataxia. The MRI results therefore cannot be seen as diagnostic of SCAIS and may just have shown mild anatomic variants of no clinical significance. Cisternal cerebrospinal fluid (CSF) analysis was normal (in four tested dogs) and CSF PCRs for canine distemper virus, *Toxoplasma gondii* and *Neospora caninum* were negative (in two tested dogs). Brain stem auditory evoked response testing was normal (in four tested dogs).

### Genetics investigations

Using a genome-wide homozygosity mapping approach with six Italian Spinoni diagnosed with SCAIS and six controls IS, we identified two microsatellite markers, C20.374 and Ren124F16, that showed a pattern suggestive of linkage to chromosome 20 of the canine genome. All SCAIS cases were homozygous and all controls heterozygous for a two marker haplotype (Table 1).

**Table 1:** Homozygosity mapping results suggesting linkage of SCAIS to CFA20. Markers C20.374 and Ren124F16 gave results suggestive of linkage to SCAIS

(A)							
Marker	Position	Case ID number					
	(Chromosome: Mb)	5357	5397	5404	6422	6477	6685
C20.374	20:15.60	191/191	191/191	191/191	191/191	191/191	191/191
Ren124F16	20:21.93	233/233	233/233	233/233	233/233	233/233	233/233

(B)							
Markers	Position	Control ID number					
	(Chromosome: Mb)	5297	5298	5405	5407	5436	6478
C20.374	20:15.60	187/191	191/191	182/191	182/191	191/191	191/191
Ren124F16	20:21.93	233/238	238/240	233/233	233/233	233/238	233/238

(A) All cases are homozygous for the same shared two marker haplotype. (B) Genotypes for control individuals

**Fig. 1:**
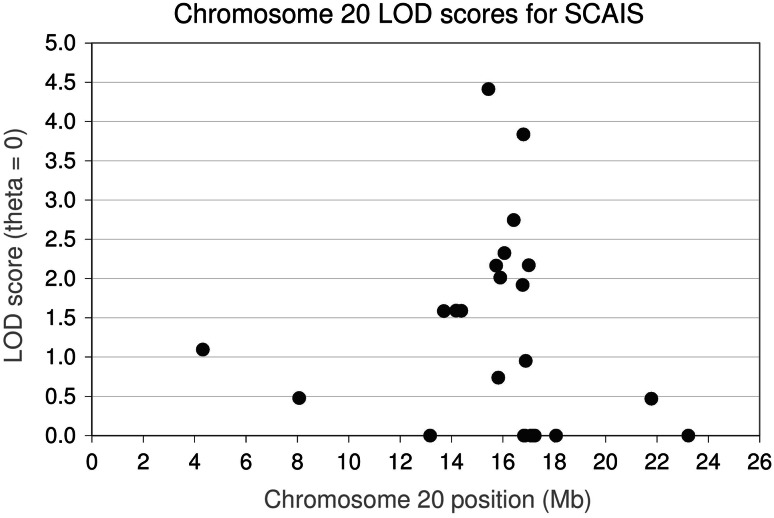
*Plot* of chromosome 20 LOD scores associated with CFA20 markers when genotyped across an extended IS pedigree consisting of 13 SCA cases and 47 controls. The maximal LOD score was 4.41 at 15.60 Mb

Linkage analysis across an extended pedigree containing 13 cases and 47 controls, including 16 obligate carriers, was performed to confirm the association of SCA to chromosome 20. Data from 22 microsatellite markers across chromosome 20 were analysed using the MLINK software package, with marker C20.374 achieving a maximal logarithm of odds (LOD) score of 4.41 at θ 0 (Fig. 1), confirming linkage.

Upon confirmation of linkage to chromosome 20 fine mapping was performed using additional markers to define the disease-associated haplotype. The 13 SCAIS cases were genotyped across a total of 40 microsatellite and 8 SNP markers, defining the disease associated

interval as chr20:15,601,140–17,116,778 based on the genomic coordinates of the CanFam2 assembly (Supplementary File 1). The disease-associated interval contained 5 genes: *BHLHE40*, *ITPR1*, *SUMF1*, *SETMAR* and *LRRN1*. All five genes were exon resequenced, although no polymorphisms were identified which segregated consistently with disease status.

**Fig. 2:**
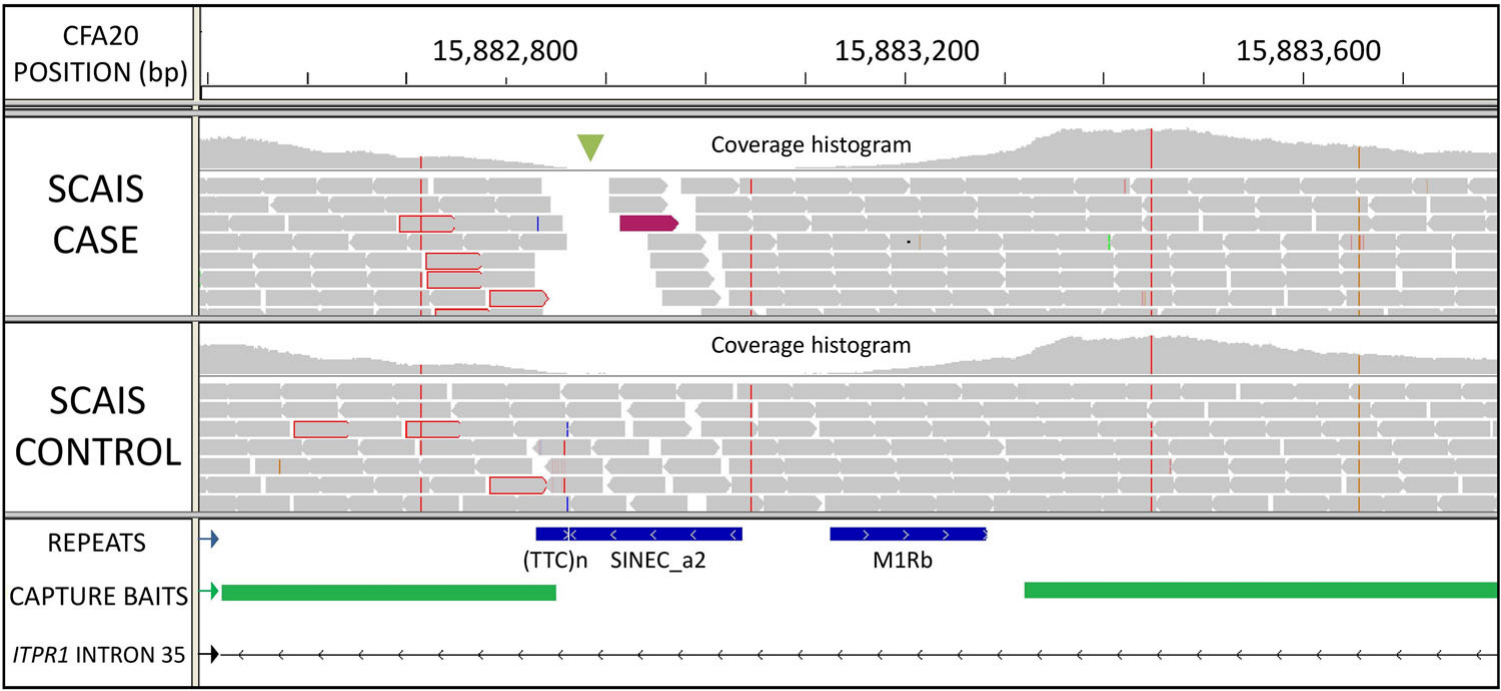
View in IGV of reads aligned to intron 35 of canine *ITPR1* for one case and one control individual. The *green triangle* indicates the position where coverage is only achieved for the control individual but not the SCAIS case. Reads (*grey bars*) with a red perimeter indicate singleton reads. Unaligned mates of singleton reads are of the sequence GAA_(17)_, suggesting repeat expansion (reference sequence GAA_(8)_) (Color figure online)

A massively parallel sequencing experiment was subsequently undertaken, using a probe-based target enrichment methodology to capture the entire disease-associated region of two SCAIS cases and three controls. Controls with haplotypes similar to the disease-associated haplotype were selected to reduce the number of potentially causal variants. A 20 Gb dataset of 51 bp paired-end reads was produced. Across the 1.52 Mb disease-associated interval a total of 3,871 SNPs and 622 INDELs were identified, although only eight of these variants segregated in accordance with disease status. All eight segregating variants could be ruled out by genotyping additional control individuals. On visual scanning of the sequence read alignments, a 30 bp region showing variable coverage between cases and controls was identified (Fig. 2). In this region, read depth dropped to zero for cases but remained in all three controls, although the profile of the coverage histogram is comparable for all individuals. Most of the unsequenced region, which was located in intron 35 of *ITPR1*, consisted of a poly(T) tract 5′ of a SINE element and 3′ of a short trinucleotide repeat sequence TTC_(8)_. Just upstream of the region many reads were present which had unaligned mates (singleton reads). Further investigation revealed the sequence of the unaligned mates to be GAA_(17)_ suggesting expansion of the trinucleotide repeat sequence.

**Fig. 3:**
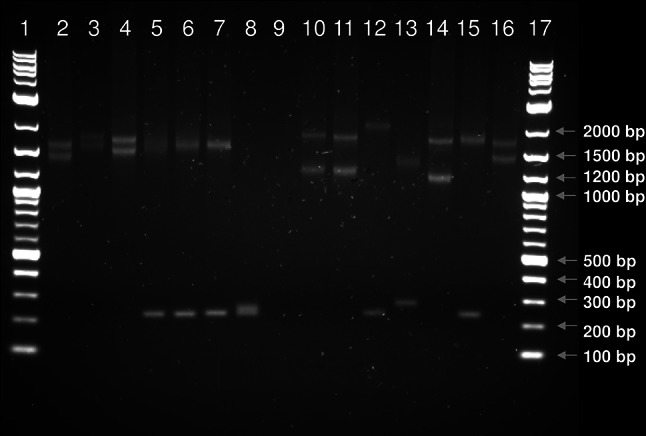
PCR analysis of the GAA repeat polymorphism. The expected wild-type allele size of the amplicon crossing the ITPR1 GAA triplet repeat expansion 238 bp. PCR products from cases are in lane *2*, *3*, *4*, *10*, *11*, *14* and *16*, from obligate carriers in lanes *5*, *6*, *7*, *12*, *13* *14* and *15* and from a homozygous wild-type individual in lane *8*. Lane *9* contains a no-template control. All cases have two expanded alleles of *varying* *lengths*. Results suggest expanded alleles ranging from GAA_300_–GAA_650_

**Fig. 4:**
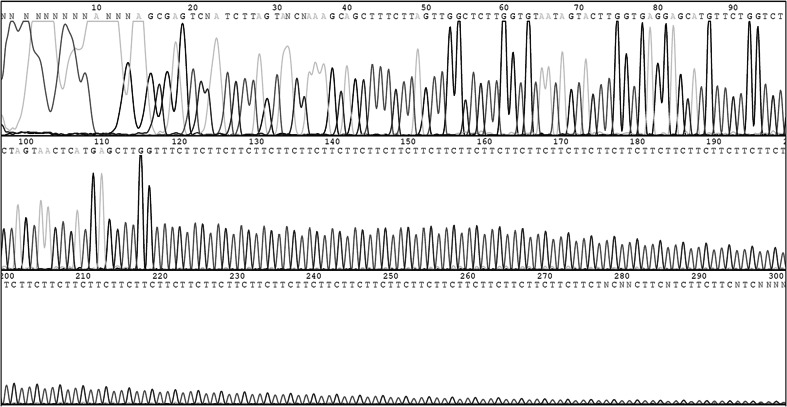
Sanger sequencing trace of the GAA (CTT) repeat expansion. Sanger sequencing of an expanded amplicon produced from a SCA case confirmed a pure GAA repeat expansion, although a complete trace spanning the entire length of the GAA repeat could not be produced

Long range PCR was used to confirm expansion of the region in cases. Amplification was only achievable using highly intact genomic DNA, meaning no results could be obtained for 6 of the 13 cases for which DNA was obtained from FFPE brain tissue. The seven remaining cases all had two expanded alleles, although none of the cases had two alleles of identical length even though the cases were all homozygous for an identical disease-associated haplotype which is indicative of a founding expansion event. Allele size ranges are demonstrated in Fig. 3. Expanded alleles were estimated to range from 318 to 651 GAA repeats in length (Supplementary File 2). All 15 obligate carriers in our sample collection were heterozygous for the repeat expansion, with non-expanded alleles ranging from approximately 7–22 subunits. Both intergeneration expansion and reduction in repeat copy number were observed. The expansion was confirmed as a pure GAA repeat expansion by Sanger sequencing of a gel extracted case allele amplicon (Fig. 4).

### Histology and ITPR1 expression

**Fig. 5:**
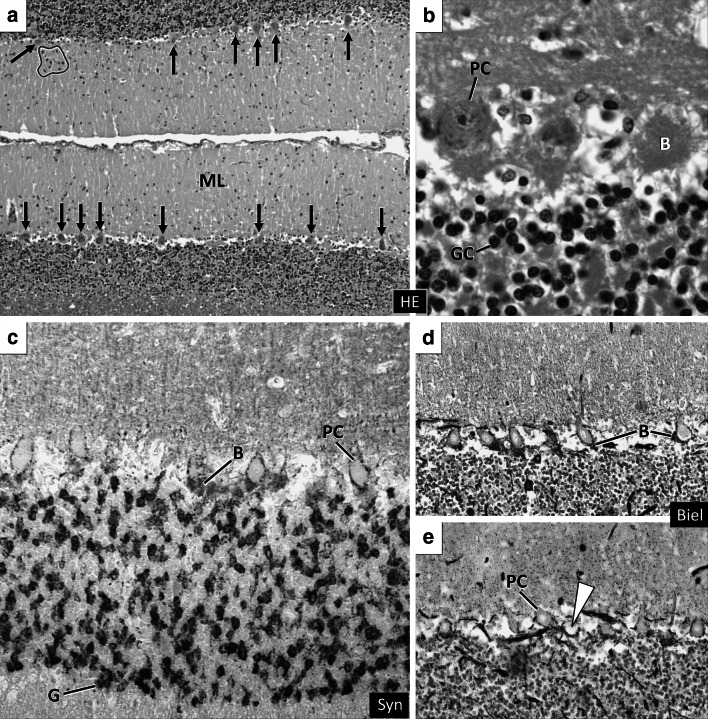
Cerebellar cortex in ITPR1 mutant IS (**a**–**c**, **e**) versus controls (**d**). Cortical layering and Purkinje cell (PC) density (**a** *black arrows*) are within a normal range. The molecular layer (ML) shows occasional foci of mildly increased stellate cell density (**a** *blue line*). Dendritic and somatic PC changes are not evident on HE slides (**a**, **b**). Instead, the baskets (*B*) appear slightly prominent (**b**) on HE. The basket cell plexus morphology, on the other hand, is normal on synaptophysin (Syn **c**) immunohistochemistry and on Bielschowsky´s stain (Biel **e**). Very rarely, empty baskets are seen in the vermis (**e** *arrowhead*). *GC* granule cell, *G* synaptic glomerulus (Color figure online)

**Fig. 6:**
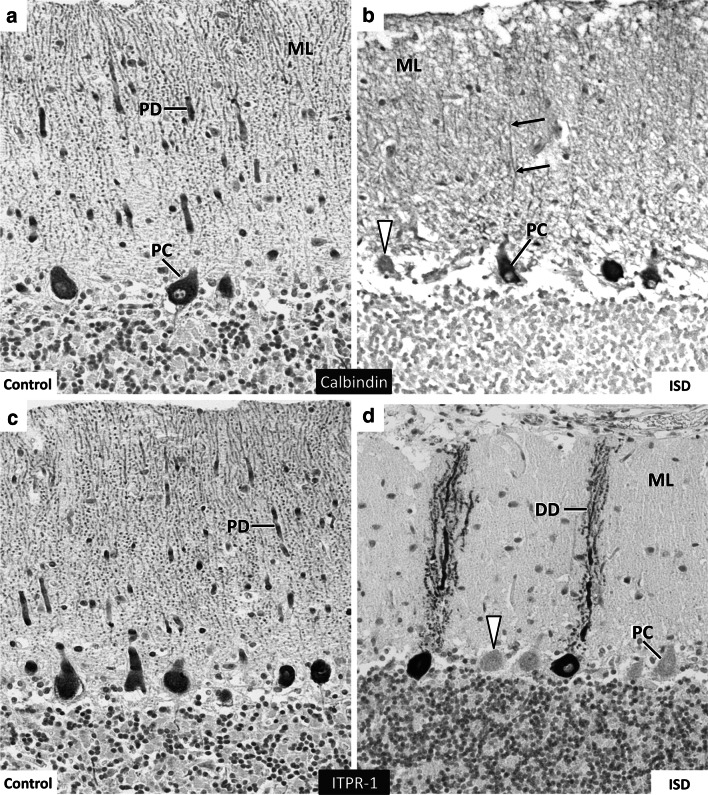
Dendritic morphology and expression of calbindin-D-28k and ITPR1 in SCA-affected IS (**b**, **d**) as compared to controls (**a**, **c**). On calbindin-D-28 k immunohistochemistry (**b**), ITPR1 mutant IS shows variable and inconstant antigen expression with scattered immunonegative PC somata (*arrowhead*) as well as with poorly stained PC dendrites (*black arrows*). A majority of about 90 % of PC stains negative or weakly positive for ITPR1 (**d** *arrowhead*). Stronger ITPR1 expression highlights the loss of planar orientation and dysmorphology of the PC dendritic tree (**d** DD). *PD* planar dendritic tree, *DD* dysmorphic dendritic tree, *ML* molecular layer

The overall size and volume ratios of ITPR1 mutant IS cerebella as well as the lobule and folia formation, the diameters of the fissures and sulci and the area of the subarachnoid space were within a normal range. Cerebellocortical layers were sharply delineated, the Purkinje cells (PC) were correctly placed and the granule cell layer presented with normal density and glomerula formation. Mild variations of the PC density (Fig. 5a) corresponded to the sublobular segmental pattern seen in age-matched control IS. In affected IS, the molecular layer exhibited some focal stellate cell hypercellularity (Fig. 5a). Dendritic and somatic changes of PC morphology were not evident. Fluorojade C accumulation was not seen in cerebellar neurons. On HE, peri-PC basket cell plexuses (BCP) appeared prominent (Fig. 5b). They stained physiologically with synaptophysin (Fig. 5c). Synaptophysin-positive halo-like structures, such as seen in human SCA-31 (Yoshida et al. 2014) were excluded. Upon Bielschowsky´s stain, BCP were sorted to type 0 to 2 at variable frequencies (Erickson-Davis et al. 2010) as they were in age-matched control dogs. Very occasionally (<1 BCP per lobule), empty baskets were detected (Fig. 5e). Even in morphologically inconspicuous PC, calbindin-D-28 k expression was inconstant and failed to highlight the dendritic morphology and orientation due to weak immunopositivity (Fig. 6b). Only a minority of PC (10–15 %) expressed ITPR1 strongly, with additional discrepancies regarding perikaryal versus dendritic staining. In control dogs, 100 % of Purkinje cells show strong ITPR1 expression throughout all lobules. Areas of ITPR1 expression in cases, however, allowed for identification of a distortion of the of monoplanar orientation of the dendritic trees. Instead of the two-dimensional arborisation in sagittal plane, the dendrites, now birch-broom-like, extended into the molecular layer towards the pial membrane. Thereby, secondary and tertiary dendrites and spiny branchlets left the stem at a moderately steep angle (Fig. 6d). Both reduced ITPR1 expression and the defective planar orientation of immunopositive cells involved all lobules and functional subfields of the affected cerebella with a mild emphasis on the spinocerebellar parts of the vermis. This notably co-localised with a moderately increased GFAP immunopositivity of Bergmann glia processes in the outer molecular layer. Bergmann´s astrocytosis was not evident on HE nor GFAP stained slides. MAP2 immunohistochemstry revealed a normal functional integration of stellate cells, basket cells, Golgi cells and granular cells. The overall histological appearance of the large afferent/efferent fibre tracks, cerebellar roof and feedback nuclei was normal. Only the density of ITPR1 positive presynapses appeared reduced in the cerebellar roof nuclei and vestibular nuclei.

## Discussion

We have identified a GAA repeat expansion in intron 35 *ITPR1* which is strongly associated with autosomal recessive SCAIS. Cases in the study had expanded alleles in the range of approximately GAA_(300)_ to GAA_(650)_ in comparison to a wild-type range of GAA_(7)_ to GAA_(22)_. All cases shared a common disease-associated haplotype, but had two expanded repeat alleles of varying length, implying that repeat length is unstable and generational expansion and contraction was observed.

The GAA expansion discovered in the IS is similar to the repeat expansion causing Friedreich ataxia in humans (Campuzano et al. 1996). Both expansions stem from a short GAA repeat on the edge of a SINE element, and result in autosomal recessive ataxic conditions. The intronic GAA repeat expansion causing Friedreich ataxia is located in intron one of the frataxin (*FXN*) gene, and results in reduced expression of the FXN transcript, potentially due to the formation of a triplex DNA structure and “sticky DNA” (Sakamoto et al. 1999).The mechanism by which reduced frataxin, a mitochondrial protein involved in iron-sulphur cluster regulation, causes neurodegeneration is debated, but recent research support a neuroinflammatory mechanism (Adinolfi et al. 2009; Hayashi et al. 2014). Triplet repeat copy number in Friedreich ataxia patients has been reported to range between 66 and 1,700 subunits, thus the GAA repeat expansion observed in canine *ITPR1* falls within this range (Durr et al. 1996; Epplen et al. 1997). Like the repeat expansion event in the IS there is evidence that Friedrich ataxia expansion alleles have a common founder (Cossee et al. 1997). Furthermore, large normal alleles also exist, which have arisen from a single founding chromosome. These large normal alleles in frataxin represent a pre-mutation reservoir and catastrophic single generation normal range to pathogenic repeat range changes have been observed (Cossee et al. 1997). This evidence suggests that although this form of cerebellar ataxia has been eradicated in the IS through a selective breeding programme, expansion of a large normal allele is possible, which could potentially cause a resurgence of the disease within the breed population. It also raises the possibility that a similar repeat expansion could be the cause of ataxia in other dog breeds.

The gene *ITPR1* encodes inositol 1,4,5-trisphosphate receptor type 1, which is an intracellular inositol tri-phosphate (IP3)-gated calcium channel, which regulates the release of calcium from the cytoplasm from intracellular stores. Signalling through IP3 and calcium release is involved in a number of cellular processes including cell division, synaptic transmission, gene expression and apoptosis (Foskett 2010). Heterozygous deletion of the *ITPR1* gene has been shown to cause SCA15 (also designated SCA16) in humans (Huang et al. 2012). SCA15 is an autosomal dominant pure cerebellar ataxia with a reported prevalence of 1.8 % of patients affected by SCA (Marelli et al. 2011) and an onset age ranging from 10 to 66 years (Miyoshi et al. 2001; Storey et al. 2001). Using EBV immortalised lymphocytes, *ITPR1* protein levels were shown to be considerably lower in cell lines derived from affected individuals, and haploinsufficiency had been suggested as the cause of the disease. Mice lacking *ITPR1* displays a severe phenotype, although most die in utero. Surviving mice show normal behaviour at birth with signs of ataxia apparent at day nine. Tonic or tonic/clonic seizures develop at day 20–23 and mice die by the weaning period (Matsumoto et al. 1996). A similar but less severe phenotype is displayed by the opt mouse, which has an in-frame deletion of exons 43 and 44 of *ITPR1* (Street et al. 1997). Extracerebellar signs may be due to lesions in other ITPR1 rich brain regions such as the hippocampal CA1 segment, basal nuclei and thalamus in knock-out mice. Human SCA15 and other genetic ataxias that imply perturbation of ITPR1-signalling pathways, such as due to *CACNA1A*, *PKCG* and *CACN4B* mutation, in contrast, presents mostly as monosystem disease with pure rather than complex cerebellar dysfunction. Also for SCAIS, extracerebellar signs have not been identified. The ataxia, moreover, is less severe than in the mouse, due to incomplete loss of *ITPR1* expression.

As in human SCA15, *ITPR1* expression is partially impaired in homozygous IS, explaining the attenuated phenotype if compared to the mouse. Disruption of ITPR1-dependent signalling is thought to affect long-term depression and the heterosynaptic plasticity in Purkinje cells with severe effects on motor coordination and learning as well as on maturation of PC dendrite morphology and synaptogenesis (Schorge et al. 2010). Moreover, the histological evidence of degenerative features and reactive astrogliosis in the IS brains offers the possibility of phenotypic worsening by premature drop out of functional PC. Current reports on ITPR1 mouse mutants failed to document changes other than the reduction of ITPR1 immunoreactivity amongst PC (van de Leemput et al. 2007). The authors do not comment on PC cytomorphology or cerebellocortical architecture. In the same vein, there are no data available on cerebellar histomorphology in SCA15 patients. Hence, *ITPR1* mutant IS may help to increase our understanding of the morphological substrate underlying this type of hereditary ataxia.

Unlike human SCA15 cases, however, ataxia has not been observed in heterozygous dogs, perhaps because more *ITPR1* transcript is produced than in haploinsufficient human cases. Alterative explanations include insufficient canine longevity to allow disease progression or failure of owners to notice phenotypic changes which may be more subtle in quadrupeds.

Identification of an intronic causal mutation highlights the limitations of using popular enrichment techniques such as exome capture for targeted resequencing as a means of mutation identification. Although the intronic GAA repeat expansion was identified using target enrichment of the disease-associated interval, mutation identification was actually facilitated by repeat masking preventing capture probes (baits) being designed across the repeat expansion location. In controls sufficient flanking sequence was captured by baits to result in sequence coverage all the way through the repetitive region. For cases, however, because the repeat expansion increased the size of the masked repetitive region, no DNA fragments were captured that were of sufficient size to span across the GAA repeat. This resulted in a region of zero coverage in cases 3′ of the GAA repeat sequence, as no probes 3′ of the GAA repeat were located close enough to capture this region. The gap in coverage in cases, but not controls provided a discernable difference that could be followed up allowing mutation identification. We speculate that the repeat expansion may also have been difficult to identify using a whole-genome sequencing approach due to the intronic positioning and because complete coverage would be achieved for both cases and controls, although an increase in singleton reads would still be observed across the region for IS cases. The repetitive nature and intronic location of the associated variant made identification challenging, with the study taking several years to complete, hence the range of classic and more modern techniques used.

To conclude, we have identified a repeat expansion in intron 35 of *ITPR1* leading to impaired protein expression in PC which is strongly associated with SCAIS. No repeat expansions in *ITPR1* have been associated with cerebellar ataxia previously and this is the first reported naturally occurring pathogenic intronic repeat expansion in a non-human species.

## Electronic supplementary material

Below is the link to the electronic supplementary material.

Supplementary material 1 (PDF 19 kb)

Supplementary material 2 (PDF 61 kb)
