# Supplementary material for: Spinocerebellar ataxia in the Italian Spinone dog is associated with an intronic GAA repeat expansion in ITPR1
Source: Mamm Genome. 2014 Oct 30;26(1):108–17. doi: 10.1007/s00335-014-9547-6 (PMC4305091; doi:10.1007/s00335-014-9547-6)
Supplement: Supplementary file 1 — Supplementary material 1 (PDF 19 kb) [file 335_2014_9547_MOESM1_ESM.pdf]

Supplementary File 1

Fine mapping genotyping results across the disease-associated region for cases and obligate carriers. Heterozygous genotypes are highlighted in blue. The disease-associated interval is defined by recombination events resulting in loss of the shared homozygosity across cases and is marked by the orange lines. Individuals 5404 and 2275 were also genotyped on the Illumina CanineHD array to assess the disease-associated interval for potential copy number variation using the logR values generated. Marker BICF2P939857 from the array genotyping defines the 3' boundary of the disease-associated interval. Note, there are some missing genotypes due to attempted PCR using DNA from FFPE tissue.

CASES

MARKER	CHR	POSITION (Mb)	a4489		a4490		a4491		a4914		a4950		a4951		a5357		a5397		a5404		a6422		a6477		a6685		a8637	
PEZ19	20	4.47	-	-	-	-	-	-	-	-	-	-	-	-	200	196	200	192	200	192	200	200	200	200	200	200	-	-
CFA20_8.07	20	8.22	-	-	-	-	-	-	-	-	276	276	-	-	276	276	276	276	276	276	276	276	276	276	276	276	-	-
C20_12.62	20	12.77	-	-	205	205	205	207	-	-	205	215	205	205	205	215	205	205	205	205	205	205	205	205	205	205	205	215
CFA20_13.17	20	13.32	-	-	247	247	-	-	-	-	247	245	247	247	247	245	247	247	247	247	247	247	247	247	247	247	-	-
C20_13.53	20	13.68	-	-	204	204	204	204	-	-	204	212	204	204	204	212	204	204	204	204	204	204	204	204	204	204	204	212
CFA20_13.70	20	13.85	-	-	240	240	240	240	-	-	240	240	240	240	240	240	240	240	240	240	240	240	240	240	240	240	240	240
CFA20_14.18	20	14.33	208	208	208	208	208	208	-	-	208	208	208	208	208	208	208	208	208	208	208	208	208	208	208	208	208	208
CFA20_14.27	20	14.42	-	-	279	279	-	-	-	-	279	279	279	279	279	279	279	279	279	279	279	279	279	279	279	279	279	279
CFA20_14.34	20	14.49	-	-	203	203	203	203	-	-	203	207	203	203	203	203	203	203	203	203	203	203	203	203	203	203	203	203
CFA20_14.38	20	14.53	-	-	134	134	134	134	-	-	134	134	134	134	134	134	134	134	134	134	134	134	134	134	134	134	-	-
C20_14.44	20	14.59	-	-	230	230	230	230	-	-	230	230	230	230	230	230	230	230	230	230	230	230	230	230	230	230	230	230
CFA20_15.03	20	15.18	-	-	162	162	162	162	-	-	162	157	162	162	162	162	162	162	162	162	162	162	162	162	162	162	162	162
CFA20_15.13	20	15.28	-	-	131	131	131	131	-	-	131	141	131	131	131	131	131	131	131	131	131	131	131	131	131	131	131	131
CFA20_15.27	20	15.42	-	-	261	261	261	261	-	-	261	267	261	261	261	261	261	261	261	261	261	261	261	261	261	261	261	261
CFA20_15.37	20	15.52	-	-	239	239	239	239	-	-	239	233	239	239	239	239	239	239	239	239	239	239	239	239	239	239	239	239
CFA20_15.39	20	15.54	-	-	167	167	167	167	-	-	167	169	167	167	167	167	167	167	167	167	167	167	167	167	167	167	167	167
C20.374	20	15.59	-	-	191	191	191	191	-	-	191	187	191	191	191	191	191	191	191	191	191	191	191	191	-	-	191	191
CFA20_15.59	20	15.74	-	-	285	285	-	-	-	-	285	285	-	-	285	285	285	285	285	285	285	285	285	285	285	285	285	285
CFA20_15.60	20	15.75	-	-	204	204	204	204	-	-	204	204	204	204	204	204	204	204	204	204	204	204	204	204	204	204	204	204
CFA20_15.74	20	15.89	-	-	177	177	177	177	-	-	177	177	177	177	177	177	177	177	177	177	177	177	177	177	177	177	177	177
CFA20_15.82	20	15.97	-	-	183	183	-	-	-	-	-	-	183	183	183	183	183	183	183	183	183	183	183	183	183	183	-	-
CFA20_15.90	20	16.05	-	-	-	-	-	-	-	-	-	-	-	-	328	328	328	328	328	328	328	328	328	328	328	328	-	-
CFA20_16.06	20	16.21	-	-	246	246	246	246	-	-	246	246	246	246	246	246	246	246	246	246	246	246	246	246	246	246	-	-
CFA20_16.42	20	16.57	-	-	-	-	-	-	-	-	-	-	-	-	164	164	164	164	164	164	164	164	164	164	164	164	-	-
C20_16.76	20	16.91	-	-	224	224	224	224	-	-	224	224	224	224	224	224	224	224	224	224	224	224	224	224	224	224	224	224
CFA20_16.77	20	16.92	-	-	155	155	155	155	-	-	155	155	155	155	155	155	155	155	155	155	155	155	155	155	155	155	155	155
CFA20_16.80	20	16.95	-	-	201	201	201	201	-	-	201	201	201	201	201	201	201	201	201	201	201	201	201	201	201	201	-	-
CFA20_16.82	20	16.97	-	-	-	-	-	-	-	-	-	-	-	-	299	299	299	299	299	299	299	299	299	299	299	299	-	-
CFA20_16.86	20	17.01	-	-	-	-	-	-	-	-	-	-	-	-	315	315	315	315	315	315	315	315	315	315	315	315	-	-
CFA20_16.89	20	17.04	-	-	-	-	-	-	-	-	-	-	-	-	289	289	289	289	289	289	289	289	289	289	289	289	-	-
BICF2P939857	20	17.12	-	-	-	-	-	-	-	-	-	-	-	-	-	-	-	-	T	C	-	-	-	-	-	-	-	-
SNP8	20	17.14	-	-	-	-	-	-	-	-	-	-	-	-	-	-	-	-	C	G	-	-	-	-	C	C	-	-
SNP7	20	17.14	-	-	-	-	-	-	-	-	-	-	-	-	-	-	-	-	T	C	-	-	-	-	-	-	-	-
SNP6	20	17.14	-	-	-	-	-	-	-	-	-	-	-	-	-	-	-	-	A	G	-	-	-	-	-	-	-	-
SNP5	20	17.14	-	-	-	-	-	-	-	-	-	-	-	-	-	-	-	-	T	C	-	-	-	-	-	-	-	-
SNP4	20	17.15	-	-	-	-	-	-	-	-	-	-	-	-	-	-	-	-	G	A	-	-	-	-	-	-	-	-
CFA20_17.01	20	17.16	-	-	-	-	-	-	-	-	-	-	-	-	320	320	320	320	320	320	320	320	320	320	320	320	-	-
SNP3	20	17.19	-	-	-	-	-	-	-	-	-	-	-	-	-	-	-	-	T	C	-	-	-	-	-	-	-	-
SNP2	20	17.19	-	-	-	-	-	-	-	-	-	-	-	-	-	-	-	-	T	C	-	-	-	-	-	-	-	-
SNP1	20	17.19	-	-	-	-	-	-	-	-	-	-	-	-	-	-	-	-	G	T	-	-	-	-	-	-	-	-
c20_17.09	20	17.24	-	-	-	-	-	-	-	-	-	-	-	-	245	245	245	245	245	237	245	245	245	245	245	245	245	245
CFA20_17.16	20	17.31	-	-	132	132	132	132	-	-	-	-	132	132	132	134	132	132	132	134	132	132	132	132	132	132	-	-
CFA20_17.24	20	17.39	-	-	297	297	-	-	-	-	297	297	297	297	297	297	297	297	297	303	297	297	297	297	297	297	-	-
CFA20_18.07	20	18.22	129	129	129	129	129	129	-	-	129	129	129	129	129	139	129	129	129	135	129	129	129	129	129	129	-	-
CFA20_19.38	20	19.53	-	-	-	-	293	293	-	-	-	-	293	293	293	293	293	293	293	293	293	293	293	293	293	293	293	293
CFA20_20.20	20	20.35	-	-	280	280	-	-	-	-	280	280	280	280	280	275	280	280	280	286	280	280	280	280	280	280	280	275
CFA20_21.28	20	21.43	-	-	162	162	162	162	-	-	162	162	162	162	162	166	162	162	162	168	162	162	162	162	162	162	162	166
Ren124F16	20	21.93	-	-	-	-	-	-	-	-	-	-	-	-	233	233	233	233	233	233	233	233	233	233	233	233	-	-
CFA20_23.22	20	23.37	-	-	-	-	-	-	-	-	271	271	271	271	271	273	271	271	271	275	271	271	271	271	271	271	-	-

OBLIGATE CARRIERS

MARKER	CHR	POSITION (Mb)	c2224		c2228		c2236		c2247		c2248		c2255		c2275		c2524		c5297		c5298		c5405		c5407		c5436		c6478		c6479		c8636	
PEZ19	20	4.47	-	-	-	-	-	-	-	-	-	-	-	-	192	200	-	-	184	196	192	200	192	204	204	200	196	196	196	200	192	200	-	-
CFA20_8.07	20	8.22	-	-	-	-	-	-	-	-	-	-	-	-	276	276	-	-	273	276	273	276	276	276	-	-	273	276	278	276	278	276	-	-
C20_12.62	20	12.77	201	205	201	215	215	205	201	205	205	205	-	-	215	205	215	205	211	205	209	205	215	205	215	205	209	215	209	205	205	205	201	205
CFA20_13.17	20	13.32	-	-	-	-	-	-	-	-	-	-	-	-	245	247	-	-	251	255	257	247	249	247	249	247	245	257	257	247	243	247	-	-
C20_13.53	20	13.68	204	208	208	212	212	204	208	204	208	204	-	-	212	204	212	204	208	208	208	204	208	204	208	204	208	212	208	204	213	204	208	204
CFA20_13.70	20	13.85	238	240	238	240	-	-	238	240	240	240	-	-	240	240	240	240	236	240	236	240	240	240	240	240	236	240	236	240	240	240	238	240
CFA20_14.18	20	14.33	208	208	211	208	208	208	208	208	203	208	208	208	208	208	208	208	203	208	212	208	212	208	212	208	212	208	212	208	208	208	211	208
CFA20_14.27	20	14.42	279	279	281	279	279	279	279	279	279	279	279	279	279	279	279	279	279	279	281	279	279	279	279	279	281	279	281	279	279	279	281	279
CFA20_14.34	20	14.49	203	203	207	186	207	203	203	203	186	203	203	203	207	203	207	203	186	203	186	203	203	203	203	203	186	203	186	203	203	203	186	203
CFA20_14.38	20	14.53	-	-	-	-	-	-	-	-	-	-	-	-	134	134	-	-	134	134	134	134	134	134	134	134	134	134	134	134	134	134	-	-
C20_14.44	20	14.59	209	230	234	230	230	230	209	230	230	230	-	-	230	230	230	230	230	230	234	230	236	230	236	230	234	230	234	230	230	230	234	230
CFA20_15.03	20	15.18	162	162	157	162	157	162	162	162	162	162	-	-	157	162	157	162	162	162	162	162	155	162	155	162	162	162	162	162	164	162	162	162
CFA20_15.13	20	15.28	131	139	139	141	141	131	139	131	139	131	-	-	141	131	141	131	139	131	139	131	133	131	133	131	139	131	139	131	137	131	139	131
CFA20_15.27	20	15.42	261	263	260	267	267	261	263	261	258	261	263	261	267	261	267	261	258	261	260	261	265	261	265	261	260	261	260	261	267	261	260	261
CFA20_15.37	20	15.52	239	243	233	237	233	239	243	239	239	239	-	-	233	239	233	239	239	239	239	239	239	239	239	239	237	239	237	239	245	239	237	239
CFA20_15.39	20	15.54	161	167	165	169	169	167	161	167	159	167	161	167	169	167	169	167	159	167	-	-	160	167	160	167	165	167	165	167	169	167	165	167
C20.374	20	15.59	186	191	186	191	-	-	186	191	186	191	-	-	187	191	186	191	187	191	191	191	182	191	182	191	191	191	191	191	192	191	191	191
CFA20_15.59	20	15.74	285	291	285	285	285	285	291	285	291	285	291	285	285	285	285	285	291	285	285	285	294	285	294	285	285	285	285	285	285	285	285	285
CFA20_15.60	20	15.75	200	204	200	204	196	204	200	204	196	204	-	-	196	204	196	204	196	204	200	204	196	204	196	204	200	204	200	204	204	204	200	204
CFA20_15.74	20	15.89	168	177	179	177	-	-	168	177	173	177	-	-	175	177	175	177	173	177	179	177	171	177	171	177	179	177	179	177	177	177	179	177
CFA20_15.82	20	15.97	181	183	192	183	183	183	181	183	188	183	188	183	183	183	183	183	187	183	191	183	189	183	189	183	191	183	191	183	183	183	-	-
CFA20_15.90	20	16.05	-	-	-	-	-	-	-	-	-	-	-	-	328	328	-	-	330	328	326	328	330	328	330	328	326	328	326	328	324	328	-	-
CFA20_16.06	20	16.21	246	248	241	246	248	246	248	246	246	246	244	246	249	246	248	246	246	246	243	246	243	246	243	246	243	246	243	246	246	246	-	-
CFA20_16.42	20	16.57	-	-	-	-	-	-	-	-	-	-	-	-	164	164	-	-	164	164	168	164	176	164	176	164	168	164	168	164	164	164	-	-
C20_16.76	20	16.91	220	224	210	224	224	224	220	224	210	224	-	-	224	224	224	224	210	224	210	224	228	224	228	224	210	224	210	224	226	224	210	224
CFA20_16.77	20	16.92	155	155	157	155	157	155	155	155	151	155	151	155	158	155	157	155	151	155	158	155	166	155	166	155	158	155	158	155	158	155	157	155
CFA20_16.80	20	16.95	-	-	-	-	-	-	-	-	-	-	-	-	199	201	-	-	192	201	201	201	205	201	205	201	201	201	201	201	201	201	-	-
CFA20_16.82	20	16.97	-	-	-	-	-	-	-	-	-	-	-	-	296	299	-	-	294	299	296	299	299	299	299	299	296	299	296	299	296	299	-	-
CFA20_16.86	20	17.01	-	-	-	-	-	-	-	-	-	-	-	-	315	315	-	-	322	315	-	-	318	315	318	315	315	315	315	315	315	315	-	-
CFA20_16.89	20	17.04	-	-	-	-	-	-	-	-	-	-	-	-	289	289	-	-	281	289	289	289	289	289	289	289	289	289	289	289	289	289	-	-
BICF2P939857	20	17.12	-	-	-	-	-	-	-	-	-	-	-	-	T	C	-	-	-	-	-	-	-	-	-	-	-	-	-	-	-	-	-	-
SNP8	20	17.14	-	-	-	-	-	-	-	-	-	-	-	-	-	-	-	-	-	-	-	-	G	C	G	C	-	-	-	-	-	-	-	-
SNP7	20	17.14	-	-	-	-	-	-	-	-	-	-	-	-	-	-	-	-	-	-	-	-	T	C	T	C	-	-	-	-	-	-	-	-
SNP6	20	17.14	-	-	-	-	-	-	-	-	-	-	-	-	-	-	-	-	-	-	-	-	A	G	A	G	-	-	-	-	-	-	-	-
SNP5	20	17.14	-	-	-	-	-	-	-	-	-	-	-	-	-	-	-	-	-	-	-	-	T	C	T	C	-	-	-	-	-	-	-	-
SNP4	20	17.15	-	-	-	-	-	-	-	-	-	-	-	-	-	-	-	-	-	-	-	-	G	A	G	A	-	-	-	-	-	-	-	-
CFA20_17.01	20	17.16	-	-	-	-	-	-	-	-	-	-	-	-	322	320	-	-	320	320	320	320	320	320	320	320	322	320	322	320	322	320	-	-
SNP3	20	17.19	-	-	-	-	-	-	-	-	-	-	-	-	-	-	-	-	-	-	-	-	T	C	T	C	-	-	-	-	-	-	-	-
SNP2	20	17.19	-	-	-	-	-	-	-	-	-	-	-	-	-	-	-	-	-	-	-	-	T	C	T	C	-	-	-	-	-	-	-	-
SNP1	20	17.19	-	-	-	-	-	-	-	-	-	-	-	-	-	-	-	-	-	-	-	-	G	T	G	T	-	-	-	-	-	-	-	-
c20_17.09	20	17.24	241	245	239	245	239	245	241	245	235	245	-	-	241	245	239	245	237	245	241	245	237	245	237	245	241	245	241	245	241	245	239	245
CFA20_17.16	20	17.31	-	-	-	-	-	-	-	-	-	-	-	-	134	132	-	-	134	132	-	-	134	132	134	132	134	132	132	132	134	132	-	-
CFA20_17.24	20	17.39	-	-	-	-	-	-	-	-	-	-	-	-	297	297	-	-	297	297	303	297	303	297	303	297	303	297	303	297	297	297	-	-
CFA20_18.07	20	18.22	-	-	-	-	-	-	-	-	-	-	-	-	129	129	-	-	139	129	139	139	135	129	135	129	139	139	139	129	139	129	-	-
CFA20_19.38	20	19.53	293	293	297	293	293	293	297	293	293	293	297	293	293	293	293	293	297	293	297	293	293	293	293	293	297	293	297	293	297	293	297	293
CFA20_20.20	20	20.35	280	280	279	280	275	280	275	280	280	280	290	280	275	280	275	280	275	279	275	275	286	280	286	280	279	275	279	280	279	280	279	280
CFA20_21.28	20	21.43	162	162	162	162	162	162	160	162	162	162	162	162	162	162	162	162	166	162	166	166	168	162	168	162	166	162	162	162	162	162	162	162
Ren124F16	20	21.93	-	-	-	-	-	-	-	-	-	-	-	-	238	233	-	-	238	233	240	238	233	233	233	233	238	233	238	233	238	233	-	-
CFA20_23.22	20	23.37	-	-	-	-	-	-	-	-	-	-	-	-	273	271	-	-	269	273	275	273	275	271	275	271	273	269	269	271	269	271	-	-
