# Supplementary material for: Spinocerebellar ataxia in the Italian Spinone dog is associated with an intronic GAA repeat expansion in ITPR1
Source: Mamm Genome. 2014 Oct 30;26(1):108–17. doi: 10.1007/s00335-014-9547-6 (PMC4305091; doi:10.1007/s00335-014-9547-6)
Supplement: Supplementary file 2 — Supplementary material 2 (PDF 61 kb) [file 335_2014_9547_MOESM2_ESM.pdf]

Supplementary File 2

GAA repeat number calculations

Migration distance measurements relative to the 100 bp ladder band.

Band size (bp)	Relative migration (mm)
100	0
200	7
300	12.5
400	17.5
500	22
600	25.5
700	29
800	32
900	34.5
1000	36.5
1200	40.5
1500	45.5
2000	51
3000	58

Estimates of GAA repeat copy number based on PCR product migration.

Gel lane	ID	Allele	Migration (mm)	Calculated size (bp)	GAA copy number estimate
2	5357	1	45	1456	414
		2	48	1699	495
3	5397	1	47	1613	466
		2	49.5	1840	542
4	5404	1	46.5	1572	453
		2	48.5	1745	510
5	5405	1	9	234	7
		2	47.5	1655	480
6	5407	1	9	234	7
		2	47.5	1655	480
7	5436	1	9.5	243	10
		2	48	1699	495
8	PRT	1	9.5	243	10
		2	10.5	261	16
9	NTC	n/a	n/a	n/a	n/a
		n/a	n/a	n/a	n/a
10	6422	1	42.5	1286	357
		2	50.5	1942	576
11	6477	1	42	1256	347
		2	50	1890	559
12	6478	1	9.5	243	10
		2	52.5	2166	651
13	6479	1	11.5	280	22
		2	44.5	1420	402
14	6685	1	40.5	1169	318
		2	49.5	1840	542
15	8636	1	9.5	243	10
		2	49.5	1840	542
16	8637	1	45.5	1493	426
		2	49	1792	526

Graphs for production of a regression line equation for estimating unknown band sizes on agarose gel.

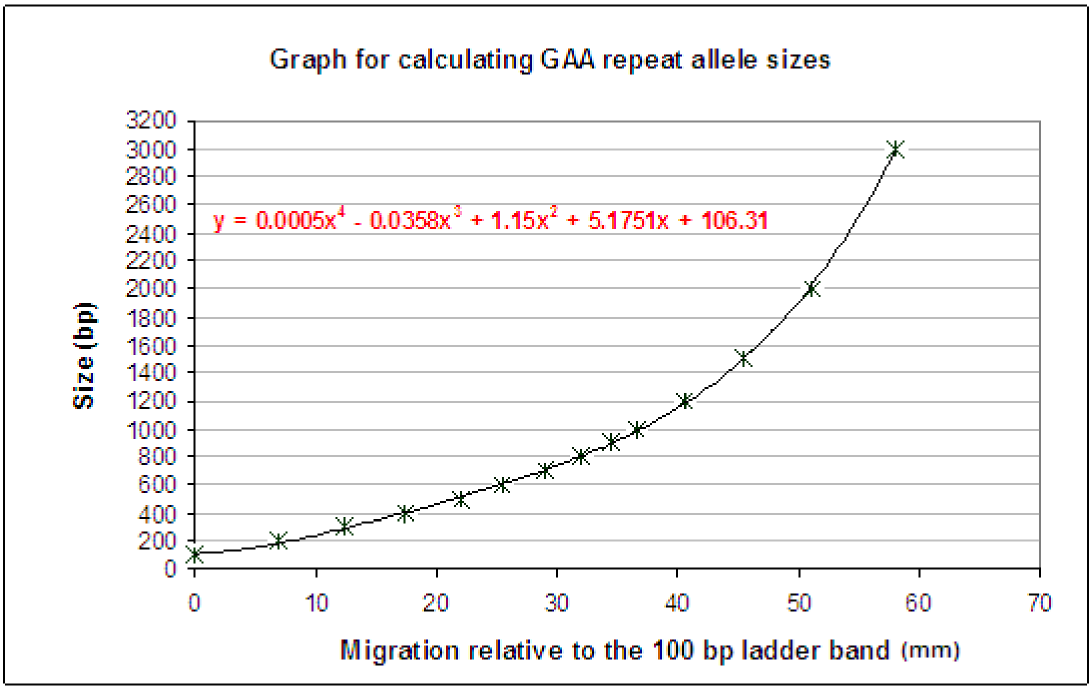

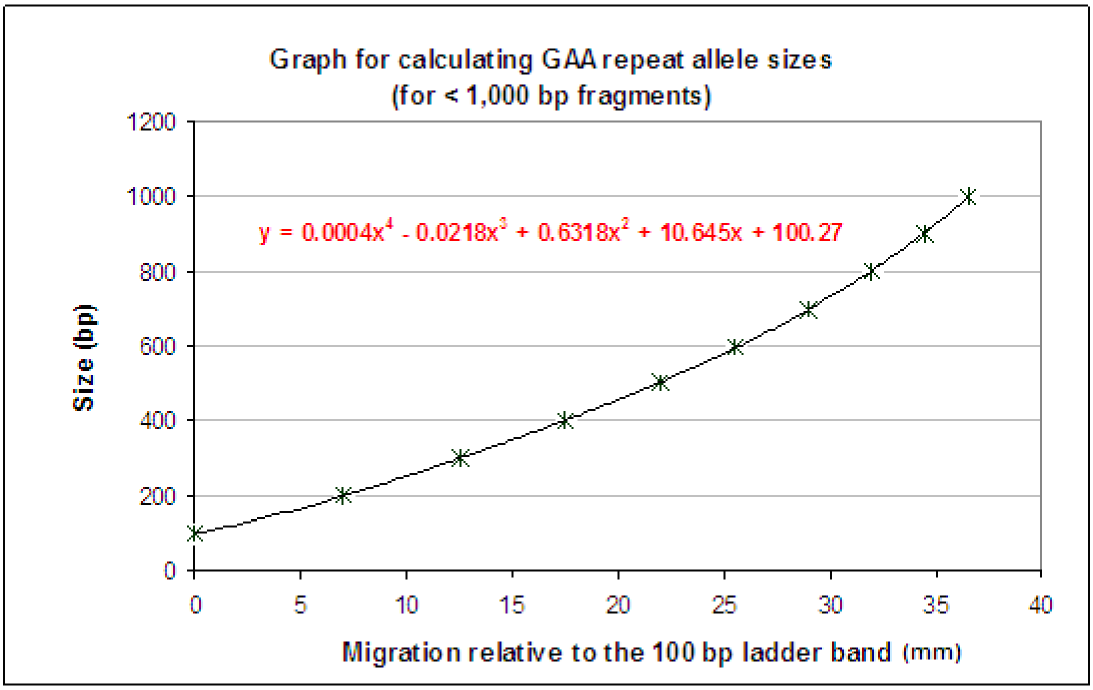

Please note the independent variable has been plotted on the y axis for optimal regression line calculation and equation purposes.

Repeat copy number and generational changes

Repeat copy number estimates based on migration distances on agarose gel. Allele sizes were calculated using polynomial regression (order 4) equations (Appendix 12). Allele copy numbers were calculated based on the reference amplicon size of 238 bp containing eight GAA repeat units.

Family	Gel Lane	ID	Relationship	Allele 1 copy number	Allele 2 copy number	Minimum generational expansion/contraction
1	7	5436	Father	10	495	n/a
	2	5357	Daughter	414	495	0
2	6	5407	Father	7	480	n/a
	5	5405	Mother	7	480	n/a
	3	5397	Daughter	466	542	+62/-14
	4	5404	Son	453	510	+30/-27
3	13	6479	Father	22	402	n/a
	12	6478	Mother	10	651	n/a
	10	6422	Son	357	576	-45/-75
	11	6477	Daughter	347	559	-55/-92
	14	6685	Son	318	542	-84/-109
4	15	8636	Father	10	542	n/a
	16	8637	Son	426	526	-16
